# Autoimmune Hemolytic Anemias: Classifications, Pathophysiology, Diagnoses and Management

**DOI:** 10.3390/ijms25084296

**Published:** 2024-04-12

**Authors:** Melika Loriamini, Christine Cserti-Gazdewich, Donald R. Branch

**Affiliations:** 1 Department of Laboratory Medicine and Pathobiology, University of Toronto, Toronto, ON M5B 1W8, Canada; melika.loriamini@mail.utoronto.ca (M.L.); christine.cserti@uhn.ca (C.C.-G.); 2 Centre for Innovation, Canadian Blood Services, Keenan Research Centre, Room 420, 30 Bond Street, Toronto, ON M5B 1W8, Canada; 3 Laboratory Medicine Program, Blood Transfusion Laboratory, University Health Network, Toronto, ON M5G 2C4, Canada; 4 Blood Disorders Program, Department of Medical Oncology and Hematology, University Health Network, Toronto, ON M5G 2C4, Canada; 5 Department of Medicine, University of Toronto, Toronto, ON M5B 1W8, Canada

**Keywords:** autoimmune hemolytic anemias, AIHA, warm-antibody AIHA, cold-antibody AIHA, PCH, direct antiglobulin test, DAT

## Abstract

Autoimmune hemolytic anemias (AIHAs) are conditions involving the production of antibodies against one’s own red blood cells (RBCs). These can be primary with unknown cause or secondary (by association with diseases or infections). There are several different categories of AIHAs recognized according to their features in the direct antiglobulin test (DAT). (1) Warm-antibody AIHA (wAIHA) exhibits a pan-reactive IgG autoantibody recognizing a portion of band 3 (wherein the DAT may be positive with IgG, C3d or both). Treatment involves glucocorticoids and steroid-sparing agents and may consider IVIG or monoclonal antibodies to CD20, CD38 or C1q. (2) Cold-antibody AIHA due to IgMs range from cold agglutinin syndrome (CAS) to cold agglutin disease (CAD). These are typically specific to the Ii blood group system, with the former (CAS) being polyclonal and the latter (CAD) being a more severe and monoclonal entity. The DAT in either case is positive only with C3d. Foundationally, the patient is kept warm, though treatment for significant complement-related outcomes may, therefore, capitalize on monoclonal options against C1q or C5. (3) Mixed AIHA, also called combined cold and warm AIHA, has a DAT positive for both IgG and C3d, with treatment approaches inclusive of those appropriate for wAIHA and cold AIHA. (4) Paroxysmal cold hemoglobinuria (PCH), also termed Donath–Landsteiner test-positive AIHA, has a DAT positive only for C3d, driven upstream by a biphasic cold-reactive IgG antibody recruiting complement. Although usually self-remitting, management may consider monoclonal antibodies to C1q or C5. (5) Direct antiglobulin test-negative AIHA (DAT-neg AIHA), due to IgG antibody below detection thresholds in the DAT, or by non-detected IgM or IgA antibodies, is managed as wAIHA. (6) Drug-induced immune hemolytic anemia (DIIHA) appears as wAIHA with DAT IgG and/or C3d. Some cases may resolve after ceasing the instigating drug. (7) Passenger lymphocyte syndrome, found after transplantation, is caused by B-cells transferred from an antigen-negative donor whose antibodies react with a recipient who produces antigen-positive RBCs. This comprehensive review will discuss in detail each of these AIHAs and provide information on diagnosis, pathophysiology and treatment modalities.

## 1. Introduction

Autoimmune hemolytic anemia (AIHA) is defined by the destruction of an individual’s own red blood cells (RBCs), caused by the existence of autoantibodies that target them [1,2,3,4,5,6,7,8]. This process leads to fewer circulating RBCs. In extreme instances, the lifespan of red blood cells (RBCs) is reduced, dropping from the usual range of 100–120 days to only a few days [1,2,3,4]. The internal components of red blood cells are released into the circulation and surrounding tissues, producing specific symptoms related to the condition [3]. Autoantibodies usually target antigens found on virtually all RBCs and a direct antiglobulin test (DAT) is useful in the diagnosis [3]. Because patients with AIHA often require transfusion to address their anemia, finding compatible blood is challenging [3,9,10]. Fortunately, transfused blood survives, as does the patient’s own, so the transfusion of antigen-selected units despite incompatibility should not be withheld [3,10]; in so doing, it is imperative to rule out underlying alloantibodies that could differentially interact with the transfused donor blood [8,9]. 

### 1.1. Acquired Hemolytic Anemias

Importantly, a positive DAT does not automatically indicate AIHA. In one study, 51% of DAT-positive patients did not have hemolysis [11]. These were predominately patients with tuberculosis and hepatitis C. 

Before one explores whether an anemic patient has AIHA, other causes of anemia must be ruled out, and evidence of a hemolytic anemia must be confirmed [3,12]. Bleeding must be ruled out as well as metabolic conditions, e.g., iron deficiency anemia or vitamin B12 deficiency. Congenital reasons for the anemia need to be ruled out, from enzymopathies (e.g., glucose 6-diphosphate (G6PD)-deficiency) to hemoglobinopathies (e.g., sickling disorders, thalassemia), and other inherited defects in structure and function (e.g., McLeod syndrome). 

Kinetic and biochemical mimics include liver cirrhosis with active gastrointestinal bleeding or resorbing large hematomas. If these are excluded, then hemolytic anemia is inferred by increases in the LDH, reticulocyte count, and (indirect) bilirubin, with the converse reduction (or complete quenching) in haptoglobin [3]. Once active, acquired hemolysis is established, the DAT is the most useful way to classify the suite of potentially explanatory AIHAs.

### 1.2. Classification and Categories of AIHA 

AIHA is classified as either primary or secondary. The disease may manifest either as a primary ailment or as a secondary condition stemming from an underlying sickness. Primary AIHA accounts for more than 60% of patients [3]. Secondary AIHA may occur due to many underlying medical disorders, including autoimmune illnesses such as lupus, chronic lymphocytic leukemia (CLL), non-Hodgkin’s lymphoma (NHL), and other blood malignancies, as well as infections caused by Epstein–Barr virus, cytomegalovirus, *Mycoplasma pneumonia*, hepatitis, and HIV [3,8,13,14]. Recently, AIHA has been associated with COVID-19 [15] AIHA is further classified according to the temperature at which red blood cells experience opsonization and destruction.

AIHA comprises seven distinct types that have been categorized mainly based on serological findings [3,12]. The seven types of AIHA are as follows: warm-antibody autoimmune hemolytic anemia (wAIHA); cold-antibody AIHA (includes cold agglutinin syndrome (CAS) and cold agglutinin disease (CAD)); mixed AIHA (also called combined cold and warm AIHA); paroxysmal cold hemoglobinuria (PCH), also called Donath–Landsteiner antibody test-positive AIHA; direct antiglobulin test-negative AIHA (DAT-negative AIHA); drug-induced autoimmune hemolytic anemia (DIAIHA); and passenger lymphocyte syndrome (PLS) (related to transplantation, and presenting as AIHA with a specificity) [2,3,5,15,16,17,18,19,20,21,22,23,24,25] (see Table 1). 

AIHA should be suspected if the patient shows signs and symptoms of anemia and other causes of the anemia, such as bleeding, underproduction, paroxysmal nocturnal hemoglobinuria (PNH), and other potential causes of anemia have been ruled out [3], see Section 1.1 above. The most important serological test for the diagnosis of AIHAs is the DAT using polyspecific and monospecific anti-human globulins (AHG) [3,12,22]. The initial testing should be with a DAT using a polyspecific AHG that contains anti-IgG and anti-C3d. If positive, then the DAT should be performed with monospecific AHG, one containing anti-IgG and one containing anti-C3d. Table 2 shows the expected results for the DAT depending on the particular type of AIHA. The antibody may only react by an indirect antiglobulin test (IAT) in wAIHA and mixed AIHA. In cold-antibody AIHA, the antibody is an IgM cold agglutinin that reacts better in colder temperatures (i.e., ≤room temperature) and involves (recruits and activates) complement so that the DAT will be positive only with polyspecific and monospecific anti-C3d AHG. In mixed AIHA, the IgG antibody reacts in the IAT while the IgM antibody is an agglutinating antibody having low titer but high thermal activity (≤37 °C). PCH will present with a positive DAT due to anti-C3d only; however, the serum autoantibody is an IgG bi-phasic hemolysin that is detected using the Donath–Landsteiner (D-L) test and recognizes the P-blood group system. Thus, it is non-reactive with p RBCs. Using standard “blood bank techniques for DAT and elution”, the DAT, eluate and serum antibody are negative in DAT-neg AIHA; however, the DAT can be shown to be positive with IgG using specialized tests such as Western immunoblotting [24]. Drug-induced AIHA serologically presents as indistinguishable from wAIHA. Diagnosis in drug-dependent forms is established when stopping the suspected drug resolves the destruction, though some drugs may trigger true wAIHA via iatrogenic immune dysregulation [3,26]. Passenger lymphocyte syndrome is diagnosed when following a solid organ or bone marrow transplant, the patient develops an antibody to the recipient’s RBCs. While presenting serologically as a wAIHA, with or without specificity, it is owing to the donor’s B-cells producing an antibody to the recipient’s residual RBCs [17,25]. 

### 1.3. Incidence 

Based on current calculations, the yearly occurrence of AIHA is 1–2 cases per 100,000 persons [18]. Out of all the instances, wAIHA is the most common type, accounting for two-thirds of all cases [3,18,19]. CAS/CAD is the second most common AIHA, accounting for 15–20% of cases [3,20]. Mixed AIHA is the third most common AIHA, as a co-presentation of both wAIHA and cold-antibody AIHA [2,3]. PCH is a rare illness disproportionately seen in children and associated with infections [3,21]. DAT-negative AIHA is rare [3,22] and difficult to diagnose [24]. Passenger lymphocyte syndrome (PLS) occurs after transplantation and is related to the immune cells from the donor, transferred via the graft, subsequently producing host-specific anti-RBC antibodies, thereby mimicking AIHA [17,25]. In the future, the incidence of AIHA may increase due to the use of stem cell transplantation and checkpoint inhibitors in the treatment of cancers [27,28]. 

### 1.4. Pathophysiology of AIHA

AIHA occurs mainly due to IgG and IgM antibodies, although, rarely, IgA antibodies may be causative [1,2,3,4,5,12,13,29]. Furthermore, complement activation is seen in all patients with CAS/CAD and the majority of patients with PCH [3,12,20]. The pathophysiology will differ based on the individual components implicated. IgG has a limited ability to activate complement and has a high affinity for the Fcγ receptor (FcγR) found on phagocytic cells [3,30]. AIHA that includes IgG can be identified by the phagocytosis of RBCs [3,31]. IgM demonstrates robust activation via the conventional complement route [3]. Therefore, AIHA characterized by IgM usually involves the destruction of red blood cells via processes involving the complement system [3]. While monocyte macrophages (MΦ) do exhibit receptors for monomeric IgM Fc, the IgG-mediated phagocytosis of red blood cells dominates, while pentameric IgM antibodies are more likely to induce phagocytosis by complement activation [3,32]. This phenomenon arises due to the presence of receptors on phagocytic cells that are specifically designed to bind to complement proteins C3b and C4b, which are detectable using a DAT with anti-complement reagents, primarily anti-C3d [3,12]. In mixed AIHA, both IgG and C3d are found on the patient’s RBCs. This is due to an agglutinating IgM cold agglutinin having a thermal amplitude to 37 °C where it is able to activate complement and a warm-reactive IgG antibody that is reactive at 37 °C causing a positive IAT [3]. In PCH, a “cold-reactive” IgG antibody binds to the patient’s RBCs at cooler temperatures (below 37 °C) and can activate complement when the temperature warms to 37 °C, though the patient’s RBCs show only residual complement (C3d) in the DAT [3]. The removal of the opsonized RBCs by MΦ are by either FcγRs if IgG has been activated or C3b/C4b receptors if complement has been activated. Usually, the removal of opsonized RBCs in AIHA with IgG antibodies takes place in the spleen, while IgG plus complement or complement alone on the RBCs occurs in the liver, specifically in Kupffer cells [3]. In rare cases, IgA may induce AIHA [3,29,33,34].

The symptoms of AIHA vary depending on the type. The symptoms may include dyspnea, fatigue, headache, muscular weakness, pallor and/or jaundice [3,8]. The development of acrocyanosis and Raynaud phenomenon in CAS/CAD may culminate rarely with gangrene [3,20]. Spherocytes are often seen in cases of wAIHA when there is the inadequate phagocytosis of antibody-coated RBCs by MΦ [3,34]. The biochemical signs of hemolysis identified in immune-mediated hemolysis include decreased hemoglobin levels, alterations in cell markers linked to hemolysis such as higher lactate dehydrogenase (LDH), reduced haptoglobin and increased unconjugated bilirubin [3,8,35]. In addition, compensatory reticulocytosis may occur in those with unsuppressed, feedback-responsive marrow reserves [3]. Some cases of AIHA occur with reticulocytopenia and the synergy of underproduction with destruction may result in severe anemia with life-threatening consequences [36,37].

## 2. Warm-Antibody Autoimmune Hemolytic Anemia (wAIHA)

wAIHA is commonly acknowledged as the primary occurrence of autoimmune-mediated hemolytic anemia [2,3,5,12,18]. Roughly half of the instances have an unknown cause, while the other half may be linked to either an existing underlying illness or the use of certain drugs [3,14,38]. Unlike cold autoimmune hemolytic anemia conditions like CAS/CAD and PCH, which are exacerbated in cold temperatures between 28 °C to 31 °C, wAIHA occurs at normal body temperature [3]. The primary antibody isotype involved with wAIHA is IgG, but IgA and IgM may be infrequently seen [3,12,29,32,33,34]. IgG antibodies have a strong attraction to red blood cells when the temperature is at the normal level of the human body (37 °C). Cold-antibody-induced hemolytic anemia, in contrast, is distinguished by antibodies that preferentially attach to red blood cells at lower temperatures, usually between 28 °C and 31 °C. This category includes CAS/CAD, PCH, and mixed AIHA. However, in mixed AIHA, the IgM cold-reactive antibody can react to 37 °C by agglutination [39]. 

The pathophysiology of the hemolysis in wAIHA is classically extravascular, mediated by the MΦ phagocytosis of antibody- and/or complement-opsonized autologous RBCs [3,26,31,35]. However, natural killer cell-mediated antibody-dependent cellular cytotoxicity (ADCC) may also be active in some cases of wAIHA [40].

Furthermore, MΦ have the capacity to selectively remove portions of the red blood cell membrane, resembling a biting action, in addition to phagocytosis [3]. This mechanism is similar to trogocytosis [41]. In wAIHA, this leads to the degradation of the RBC membrane, causing transformation into spherocytes [3,34]. Spherocytes possess less pliancy compared to regular erythrocytes and are specifically singled out for removal in the red pulp of the spleen and other constituents of the mononuclear phagocyte system. The observed expansion of the spleen, splenomegaly, is related to the trapping of spherocytes and autoantibody-opsonized RBCs in the red pulp of this organ [3].

### 2.1. Autoantibody Specificity

The specificity of autoantibodies in wAIHA has historically been tracked with the Rh blood group system, because autoantibodies that reacted with all normal RBCs would fail to react with rare RBCs lacking certain or all Rh antigens, from Rh dash-D-dash (-D-) to Rh_null_ RBCs. For example, warm autoantibodies that reacted with all normal RBCs but not with -D- and Rh_null_ RBCs were termed anti-nl (non-deleted or normal), while autoantibodies that would react with all normal RBCs and with -D- but not Rh_null_ were termed anti-pdl (partially deleted). Finally, autoantibodies that would react with normal, as well as with both -D- and Rh_null_, were termed anti-dl (fully deleted) autoantibodies [3]. Subsequently, other specificities were reported to exist; for example, anti-Jk^a^, anti-Gerbich, anti-LW and anti-Kell [3,42,43]. Other scientists found autoantibodies reacting with components of band 3 on the RBCs [3,42]. As investigators dealt with the various specificities reported and were using Rh_null_ and -D- RBCs to determine the anti-nl, anti-pdl or anti-dl specificity of these autoantibodies, two groups, independently, found that in the majority of patients with wAHIA, autoantibodies reacted preferentially with older, reticulocyte-depleted RBCs and reacted much less or not at all with younger, reticulocyte-enriched RBCs [44,45]. Both groups found that the frequency of reactivity was 80% of patients with wAIHA for the former and 20% for the latter. Thus, warm autoantibodies in 80% of the cases showed a tropism to “old RBCs” compared to “young RBCs”. One group termed this specificity as Type I wAIHA versus Type II wAIHA (20% of patients) with wAIHA showing no RBC age preference [45]. These investigators suggested that Type I wAIHA could be an exacerbation of the normal clearance mechanism in RBC senescence, whereby natural autoantibodies deplete old RBCs by targeting band 3 [45,46,47,48].

More recently, teams have shown that most warm autoantibodies do, in fact, target band 3 [42,49,50]. In a recent publication, the authors confirm Type I and Type II wAIHA and suggest that Type I wAIHA autoantibodies target band 3 and that the hemolytic anemia may conversely involve patient RBCs that are also aging faster than normal [48]. Type I patients’ RBCs, by aging faster, expose the immune system to more senescent RBC antigen, which in turn stimulates more autoantibody to senescent band 3, producing a functional autoimmune disease [42]. In contrast, patients with Type II wAIHA showed a lack of RBC aging, suggesting rapid and equal-opportunity opsonisation and the removal of RBCs [49], consistent with a previous report of Type II wAIHA being more severe than Type I wAIHA [44]. Additional investigations show that the natural autoantibody that binds to aged RBCs, indeed, recognizes band 3 [49,50]. Taken together, these findings generate the hypothesis that most wAIHA patients, at 80%, produce more of the senescent RBC autoantibody that reacts with patients’ accelerated aging RBCs, for Type I wAIHA [42,49]. Another implication from this work is that the specificity of the autoantibody in Type II wAIHA may be to other RBC antigens (non-band 3) [3,45]. These hypotheses require further verification.

### 2.2. Biomarkers

Identifying biomarkers specific to particular disease states is an ongoing quest by biomedical researchers. Although in AIHAs, the DAT is a true biomarker with classification power (Table 3), signature cytokines may also qualify as biomarkers, given specific associations in wAIHA [51,52,53,54]. In the most comprehensive report [51], 54 patients having DAT+ wAIHA were compared with 36 healthy controls for 38 cytokines, chemokines or growth factors using a multiplex approach and Luminex technology. This found that TNFα and interleukin 10 (IL-10) are the most increased in wAIHA, with IL-8/CXCL8 and IP10/CXCL10 being elevated and, hence, potentially eligible as biomarkers too. Taken together, wAIHA patients have four possible cytokine/chemokine biomarkers, with TNFα, IL-10, IL-8/CXCL8 and IP10/CXCL10, and perhaps IL-6 [53]. Future studies may validate this repertoire.

### 2.3. Treatment

wAIHA is usually initially treated with glucocorticoids [3,55,56,57,58,59]. While glucocorticoids often achieve a partial or complete remission, difficult or dependent cases may command other treatments such as off-label rituximab (anti-CD20), IVIG, or daratumumab (anti-CD38). Other investigational agents such as FcγRn inhibitors (nipocalimab), Syk kinase inhibitors (fostamatinib), BTK kinase inhibitors (rilzabrutinib), or PI3 kinase inhibitors are being explored [57,58,59,60,61,62,63,64]. If complement-mediated hemolysis is suspected, treatment may include inhibitors of the complement pathway such as sutimlimab (anti-C1s) or eculizumab (anti-C5) [58,60,63,65]. There continues to be active research on new therapies for wAIHA [58,63,64,65,66,67,68]. Although splenectomy was used for years with immense success, it is rarely used today [3,60]. In a recent cohort study of 1824 patients, splenectomy was found to be an effective treatment, though with surgical complications in 12% [69].

## 3. Cold-Antibody Autoimmune Hemolytic Anemia 

Cold-antibody autoimmune hemolytic anemia, referred to as cold agglutinin syndrome (CAS) or cold agglutinin disease (CAD), is a rare autoimmune condition characterized by the presence of high levels of cold-sensitive autoantibodies in the blood, primarily IgM, as well as autoantibodies that remain active at temperatures below 30 °C (86 °F) [3,12,20,70,71,72,73,74,75,76]. The antibodies have a particular affinity for red blood cells, often the Ii RBC antigens [3,12,77,78], causing them to aggregate (agglutination), activate the complement system, and undergo intra- and/or extravascular hemolysis [3,12,20,67,68,69,70,71,72,73,74,75,76,77]. Historically, CAD included both CAD and CAS; however, these two conditions are now recognized as different and have been separated into distinct entities. 

### 3.1. Cold Agglutinin Syndrome

Cold agglutinin syndrome (CAS) is a transient condition that is secondary to a bacterial or viral infection, such as *Mycoplasma pneumoniae* or Epstein–Barr virus causing mononucleosis [2,3,70,77]. CAS can also occur following certain malignancies and other autoimmune disorders [70,73,74,75]. CAS is also known as secondary cold agglutinin disease. CAS is self-remitting, and the condition usually resolves when the underlying infection clears [3]. 

### 3.2. Cold Agglutinin Disease

Cold agglutinin disease (CAD) or primary CAD is a clonal disease whereby the lymphoproliferation of a B-cell clone produces a cold-reactive (≤30 °C) monoclonal IgM autoantibody having specificity for the I or i RBC antigens [78]. The monoclonal IgM can activate complement and cause intravascular hemolysis. CAD is a chronic condition [73,74,75,76,78].

Both CAS and CAD have similar findings for diagnosis. Both are caused by an IgM autoantibody that targets either I or i RBC antigens. The IgM autoantibodies are naturally occurring, polyclonal anti-I or anti-i with CAS; and, in CAD there is an abnormal production of a monoclonal autoantibody, associated with a B-cell clone. The IgM autoantibody in both conditions activates complement and can cause severe intravascular hemolysis [79]. Thus, both will have a positive DAT due to only complement (C3d) on their RBCs, and both will have a cold agglutinin titer at 4 °C ≥ 64, with a thermal range ≤ 30 °C [3,12]. 

### 3.3. Treatment

Treatment for CAS usually involves keeping the patient warm until the condition resolves with convalescence from the underlying disease. Maneuvers include blanketing or maintaining higher ambient room temperatures at 37 °C–40 °C, and if transfusion is required, using a blood warmer. In severe cases of CAS, one may use sutimlimab (anti-C1s) [79,80,81,82,83,84,85,86].

CAD is a more difficult-to-treat AIHA, as the antibody is usually associated with a chronic disease and the monoclonal antibody produced can cause more severe anemia than in CAS [79,80,81,82,83,84,85,86]. Therefore, treatment can consist of rituximab or anti-complement therapies such as sutimlimab to block the classical complement activation pathway [58,65,79,80,81,82] or eculizumab to block the membrane attack complex of complement activation pathway [58,78,83,84]. To limit the production of the causal antibody and to diminish the cellular biomass of the driving condition, rituximab with or without strong immunosuppressive drugs such as fludarabine or bendamustine may be prescribed [58,78,83,84]. Recently, the use of daratumumab (anti-CD38) has been shown to be effective in the treatment of relapsing CAD [64,83,84].

## 4. Mixed AIHA (Combined Cold and Warm AIHA)

Mixed AHIA or combined cold and warm AIHA is characterized by the simultaneous presence of an IgG warm autoantibody and a cold-reactive IgM antibody with a low titer but a broad temperature range in the blood circulation, reacting up to 37 °C [2,3,39,87,88,89,90,91,92,93]. The syndrome is marked by significant hemolysis, resulting from both intravascular and extravascular hemolysis. The illness may be severe but shows a favorable response to steroid therapy [39]. However, mixed AIHA often follows a chronic trajectory with intermittent periods of deterioration [2,3,89,91]. The condition accounts for 5–8% of all AIHA; of these, 50% of cases are idiopathic while 25–42% of cases are associated with systemic lupus erythematosus (SLE) [3,4].

### Treatment

Treatment usually involves glucocorticoids with patients having a good and rapid response [39]. However, again, rituximab and/or complement inhibitors may be considered [60,91]; see treatments under wAIHA and CAD above. 

## 5. Paroxysmal Cold Hemoglobinuria or Donath–Landsteiner Test-Positive Hemolytic Anemia

Paroxysmal cold hemoglobinuria (PCH) is a rare autoimmune hemolytic anemia that is defined by the destruction of red blood cells in the blood vessels due to the activation of the complement system [2,3,94,95,96,97,98,99,100,101,102,103,104]. This destruction happens when the body is exposed to cold temperatures [3,12,100]. PCH is characterized by the presence of a cold-reactive IgG autoantibody. This antibody attaches to RBCs at temperatures below 37 °C. When the RBCs are warmed to body temperature, the antibody activates complement, and the IgG antibody subsequently dissociates from the RBCs [3,12,100]. As a result, only complement is detected on the cells. 

PCH was first documented in 1904 by Julius Donath and Karl Landsteiner, establishing it as one of the earliest recognized kinds of AIHA [3]. The illness may present either as a sudden, non-recurring post-infectious event in children [3,12,21,94,96,97,98,99,100,101,102] or as recurring occurrences in adults with blood cancers or advanced syphilis [3,20,95,96]. Historically, PCH manifested primarily in patients having syphilis, and exposure to cold resulted in paroxysms of hemoglobinuria. Today, PCH is almost always encountered as an acute transient syndrome in young children with a recent history of a viral illness [3,94,95,96], so that paroxysms resulting from cold exposure are rarely seen. Thus, it has been suggested that a better term for this condition would be Donath–Landsteiner test-positive hemolytic anemia. Another interesting finding in PCH is erythrophagocytosis by neutrophils seen in the peripheral blood [3]. Although not diagnostic, it should cue to the use of the D–L test.

### 5.1. Donath–Landsteiner Test

The diagnosis is made in the laboratory using the Donath–Landsteiner test (D–L test) [3,12,100]. This test utilizes the findings of anti-GLOB (formerly anti-P) as the biphasic IgG autoantibody, acting against RBCs positive for the GLOB antigen (formerly the P-antigen) and not RBCs lacking the GLOB antigen, GLOB-null or, formerly, p RBCs. RBCs incubate with the patient’s serum and a fresh source of complement (AB serum freshly isolated and stored frozen). The test system moves from room temperature to 37 °C. If the GLOB + RBCs are hemolyzed and the GLOB-null RBCs are not, then the D–L test is positive and the diagnosis of PCH is confirmed. 

### 5.2. Treatment

PCH in children is usually self-remitting. In severe cases requiring transfusion, approaches that have been successfully described include plasma exchange, rituximab, and complement inhibitors such as eculizumab [3,97,101,102,103,104].

## 6. DAT-Neg AIHA 

Approximately 1% to 10% of people diagnosed with AIHA have a negative DAT [3,103,104]. The DAT is a diagnostic technique that has a sensitivity range from 92% to 97% for detecting AIHA. The diagnosis of DAT-neg AIHA mostly relies on the method of excluding other possible causes [3,12,105,106,107]. Several key variables contribute to the development of DAT-neg AIHA. These factors include the following: (a) the existence of red blood cell-bound IgG at levels that are too low to be detected using standard methods [3,24,105]; (b) the relatively weak binding strength of IgG [3]; (c) the lack of a positive eluate using standard methods; and (c) the presence of RBC-bound IgA where AHG contains only antibodies to IgG [3,73] or, in rare instances, IgA or IgM [3,22,29,32,108].

In the early 1970s, Gilliland and colleagues [3,105] were the first to emphasize that the amount of antibodies found on RBCs in patients with AIHA may be insufficient to detect by standard DAT reagents. They proposed that the distribution of IgG antibodies on circulating RBCs was non-uniform. RBCs accumulate IgG as they age, leading to a larger ratio of IgG to RBCs in older RBCs compared to younger ones [44,45,49,50,105]. 

In some instances of DAT-neg AIHA, less often encountered immunoglobulins, namely IgA and IgM, may be identified on the outside of RBCs using a DAT with special antibodies [3,22,29,32,33,108]. IgA AIHA has a clinical picture that closely matches that of IgG wAIHA [106]. While the standard DAT may yield negative results, the use of special DAT reagents that can detect IgM and IgA may be useful [3]. Western immunoblotting may detect IgG on RBCs below the detection level of a DAT and could be useful to diagnose DAT-neg AIHA [24]. This technique could also be adjusted to monitor IgA and IgM levels on RBCs in DAT-neg AIHA. Most instances of IgM AIHA are caused by the presence of a pathological cold autoantibody that has a broad temperature range and may respond at temperatures ranging from 30 °C to 37 °C (see Section 3 and Section 4, above). Diagnosing warm IgM AIHA might be difficult since there are no significant serological findings. Antibody detection tests may exhibit little reactivity. Both pathogenic IgM cold autoantibodies and warm IgM autoantibodies exhibit the presence of complement coating on the patient’s RBCs. As a result of this feature, some people with warm IgM AIHA may be misdiagnosed with CAD or PCH [3,32].

### Treatment

Treatment for severe cases of DAT-neg AIHA would be similar as to those used for the treatment of wAIHA (see Section 2 above).

## 7. Drug-Induced Immune Hemolytic Anemia

Drug-induced immune hemolytic anemia (DIIHA) is a rare condition primarily caused by the existence of drug-induced antibodies, which may be classified as either drug-dependent or drug-independent [16,38,109,110,111]. Patients with DIIHA may have signs of the rapid destruction of red blood cells inside blood vessels quickly after receiving the medicine. This may be seen, for example, in babies who develop hemolytic anemia because of ceftriaxone therapy [110,111]. In contrast, some people may have less severe signs of extravascular hemolysis, which might occur many months after therapy, as shown in instances of methyldopa-induced hemolytic anemia [26,38].

### 7.1. Drug-Dependent Antibodies

Drug-dependent antibodies may be detected by analyzing RBCs that have been treated with drugs, termed the “hapten-specific mechanism”, or untreated RBCs in the presence of a drug solution, termed the “neoantigen-dependent mechanism” [38]. Both mechanisms require the drug to somehow interact with proteins on the RBCs, forming a hapten (drug)-carrier (RBC) complex which can elicit an antibody response to the drug (hapten). Examples include penicillin and cephalosporins [38,109,110]. Alternatively, a drug may complex with the RBC in vivo forming a compound antigen, a neoantigen, that requires the drug to be in the testing system to detect its activity [38]. Examples of this mechanism include second- and third-generation cephalosporins such as cefotetan and ceftriaxone [38,110,111]. 

In some instances, the antibody that is detected appears to be an autoantibody resembling autoantibodies found in wAIHA [16,26,38,111]. In this case, the DAT is positive for IgG without apparent drug-dependence and with IgG antibody detectable in the patient’s plasma [16]. The prototypical drug that causes this type of DIIHA is alpha-methyldopa [26,38]. This phenomenon has been termed the “cross-reactive autoantibody mechanism” [38]. Finally, there is an unusual mechanism whereby following certain drug therapies, the patient’s RBCs are able to take up proteins from their surroundings, including immunoglobulins, and give a positive DAT. With IgG on the patient’s RBCs, this can result in monocyte–macrophage recognition and extravascular hemolysis. This mechanism is poorly understood, but it has been suggested that it may be pH dependent [38,109].

### 7.2. Drug-Induced Autoimmune Hemolytic Anemia (DIAIHA)

There are two types of DIAIHA, cross-reactive autoantibody production and immunoglobulin adsorption. 

#### 7.2.1. Cross-Reactive Autoantibody Mechanism

The mechanism of cross-reactive autoantibody production results in an IgG warm autoantibody being made by the patient that is dependent on the drug being administered. The classic example is that of alpha-methyldopa therapy [16,38]. In these conditions of drug-induced autoimmune hemolytic anemia (DIAIHA), the patient presents with acquired hemolytic anemia with a DAT positive for IgG and negative for complement and a serum antibody that reacts with all unrelated RBCs [16,26,38]. Thus, this condition looks like classical wAIHA. It has been postulated that the drug interacts with the RBC membrane to modify it enough that it appears as “non-self” to the patient’s immune system, and this then results in the patient making an antibody to the RBCs plus the drug, but also a “crossreactive” antibody that does not require the drug [26,38]. Stopping the administration of the implicated drug will resolve the anemia; however, it may take some time for the autoantibody to go away [3,16,26]. It is unknown why only a small percentage of patients will develop this condition; however, the resulting hemolytic anemia can be severe and even life-threatening. Methyldopa is rarely used anymore. Currently, drugs that can result in the production of an IgG autoantibody to the patient’s RBCs include cefotetan and ceftriaxone [38,111]. These drugs also act by both hapten-specific and neoantigen-dependent mechanisms, making these two drugs potentially dangerous [110,111].

#### 7.2.2. Immunoglobulin Adsorption Mechanism

The other mechanism that can present as wAIHA is when drugs cause the adsorption of serum proteins onto the RBCs. This previously was termed the “nonspecific adsorption mechanism” and results in any serum protein being “adsorbed” onto the RBCs, including albumin, complement components and immunoglobulins, in particular IgG [3,109]. The patient can develop hemolytic anemia due to the IgG being on the patient’s RBCs. When the laboratory investigates the reason for the anemia, the DAT is positive for IgG. However, the eluate will be negative, providing a clue that this may be drug related. Although the implicated drug can cause the adsorption of complement components, these would be C2, C3 and C4 that normally circulate in the blood, but not the complement component that is assessed in the DAT, which is C3d. Thus, only IgG would be detected using AHG. 

Any time a patient with acquired immune hemolytic anemia is encountered with an IgG-positive DAT and a negative eluate, a drug history should be obtained, as this outcome may be related to a drug-induced hemolytic anemia. The reason for this phenomenon of immunoglobulin adsorption has been postulated to be due to the chemistry of the drugs, with some drugs having chemical structures that allows these drugs to covalently bind to both RBC proteins and external proteins in the patient’s serum [38,109]. In vitro, drugs optimally bind to RBCs depending on the pH, with a more basic pH allowing for hapten-specific drug interactions, such as with penicillin [3,16,38]. It has been shown that immunoglobulin-adsorption also occurs optimally under alkaline conditions [109]. Patients might therefore manifest this phenomenon if taking applicable drugs in the context of metabolic alkalosis.

### 7.3. Treatment

The treatment for these drug-induced conditions often simply involves stopping the drug therapy, if possible, or switching to a different, chemically unrelated, drug. When this is done, the hemolysis may abruptly cease [16,26,38]. If the hemolysis is severe, one can treat the patient with similar therapies as used for the treatment of wAIHA.

## 8. Passenger Lymphocyte Syndrome

Passenger lymphocyte syndrome (PLS) is an unusual hemolytic occurrence that can resemble AIHA. Allogeneic transplantation includes bone marrow transplantation (BMT) and solid organ transplantation. In BMT, host lymphocytes are transferred into the recipient from the bone marrow or stem cell material obtained from the donor [17,25,112,113]. In solid organ transplantation, donor lymphocytes can be “passengers” in the solid organ obtained from the donor, and these can be transferred into the recipient [114,115,116,117,118]. It is feasible for donor lymphocytes that have been transferred to produce antibodies that are specific to RBC antigens in the recipient’s body but not present on the donor’s own red cells [17,25,112,113,114,115,116,117,118]. Indeed, sometimes these passenger lymphocytes in either BMT or solid organ transplants are obtained from donors who have been sensitized to produce alloantibodies to antigens for which they lack. Thus, passenger lymphocytes from a blood group O donor transferred into a recipient of blood group A can produce anti-A that can react with and hemolyze the residual RBCs in the recipient due to those being blood group A. Likewise, passenger lymphocytes from a donor who is Rh-negative but who has been sensitized to produce anti-D can induce a hemolytic anemia in an Rh-positive recipient, which may manifest as a delayed-type hemolytic reaction. In both instances, the hemolytic anemia can appear as an AIHA, though with an apparent specificity. Indeed, if this syndrome occurs, its presentation as immune hemolysis after a transplantation may be misinterpreted as autoimmune hemolytic anemia. The hemolysis can be severe with outcomes such as renal failure [114,116,117]. 

### 8.1. Role of Cyclosporine

The specific influence of cyclosporine on the promotion of antibody production from the donor is still not fully understood. Because hemolysis has been most frequently related to cyclosporine [113], a medication that suppresses the immune system, it is suggested that donor B lymphocytes proliferated and produced antibodies because of cyclosporine effects to selectively inhibit T-cell function [117]. Alternatively, previously sensitized lymphocytes when exposed to an antigen in the presence of cyclosporine can still respond to antigens on recipient RBCs [119]. 

### 8.2. Treatment

It is critical to recognize that the stem cell or solid organ transplant patient who is hemolyzing may be manifesting PLS. Treatment may require the transfusion of antibody-evading (donor blood type) RBCs, i.e., blood group O cells if hemolysis is due to ABO antibodies (in a group O donor to a non-O recipient). If anti-D or other alloantibodies are identified as the cause of the hemolysis, then antigen-negative donor blood should be used for transfusion [17,113,115]. If hemolysis is severe, one can treat with changes to the immunosuppressive regimen [113,118] or combine the regimen with IVIG or plasmapheresis [117].

## 9. Current Opinion

We believe that hyperhemolysis syndrome (context: sickle cell disease (SCD) or other diagnoses) deserves its own category within AIHA. Although it is a distinctly transfusion-triggered event, with the absence or presence of an involved alloantibody cognate to a triggering unit, once triggered, it appears to be an autoimmune-like phenomenon with extreme bystander hemolysis [120] (kinetically akin to post-transfusion purpura (PTP)). The complement cascade appears important (as judged by the response of some cases to eculizumab), with hyperinflammation occurring (as judged by the greater responsiveness to IVIG with high-dose steroids, +/− the utility of anti-cytokine storm interventions such as tocilizumab). In virtually every regard, this is a pathology that is not addressed by any of the conventional AIHA categories. However, because it manifests in part with an autologous response, there is an argument for its inclusion in the taxonomy of AIHA, just as there are arguments for the inclusion of passenger lymphocyte syndrome. 

## 10. Summary and Perspectives

The review herein has addressed the full suite of autoimmune hemolytic anemias with its seven categories (Table 1). Within each category described, there is a discussion of clinico-laboratory features, diagnostics and management options. Treatments are tailored for each category and patient in “personalized medicine”. This is especially true if the AIHA is refractory to current first-line therapies, such as glucocorticoids, used in most AIHAs except for cold-antibody AIHAs. Existing and future options need further examination [58]. Monoclonal antibody therapies targeting B-cells using rituximab and daratubumab are showing efficacy in some cases. Targeting the complement activation pathway using sutimlimab or eculizumab is increasingly suggested in CAD and mixed AIHA [62,121]. Older therapies are also still viable in some cases, such as splenectomy and plasmapheresis. The future anticipates more monoclonals to complement components [58], the inhibition of the neonatal Fcγ receptor (FcγRn) (nipocalimab) [62], immunosuppressive combinations (mycophenolate mofetil, rapamycin) [122,123,124], kinase inhibitors (B-cell tyrosine kinase (BTK) [125], spleen tyrosine kinase (SYK) [126], Janus kinase (JAK) [127]), and proteasome inhibitors [128]. Targeting T-cell interactions with co-stimulatory molecules such as CD80/CD86 on antigen presenting cells (e.g., abatacept) shows utility [129]. In summary, despite a number of AIHAs exhibiting differential treatment response profiles, the future looks promising for mechanism-informed therapeutics.

## Figures and Tables

**Table 1:** Categories of Autoimmune Hemolytic Anemias.

Warm-antibody autoimmune hemolytic anemia (wAIHA)
Cold-antibody autoimmune hemolytic anemia
Cold agglutinin syndrome (CAS)
Cold agglutinin disease (CAD)
Mixed autoimmune hemolytic anemia (mixed AIHA)
Paroxsymal cold hemoglobinuria (PCH)
Direct antiglobulin test-negative autoimmune hemolytic anemia (DAT-neg AIHA)
Drug-induced autoimmune hemolytic anemia (DIAIHA)
Passenger lymphocyte syndrome (PLS)

**Table 2:** Serological Diagnosis of Autoimmune Hemolytic Anemias.

Category	DAT ^#^		Eluate	Serum Antibody
wAIHA	IgG *	67%	Pos (IgG)	IgG panagglutinin
	IgG + C3d	20%		
	C3d only	13%		
CAS	C3d only		Neg	Polyclonal agglutinating IgM
CAD	C3d only		Neg	Monoclona agglutinating IgM ^+^
Mixed AIHA	IgG + C3d		Pos (IgG)	IgG panagglutinin + agglutinating IgM with low titer but high therm amplitude (≤37 °C)
PCH	C3d only		Neg	Cold-reactive IgG biphasic hemolysin **
DAT-Neg AIHA	Neg ***		Neg	Neg
Drug-induced AIHA	IgG only		Pos (IgG) ^	Pos (IgG)
Passenger lymphocyte Syndrome	IgG only		Pos (igG)	IgG ^&^

wAIHA, warm-antibody autoimmune hemolytic anemia; CAS, cold agglutinin syndrome; CAD, cold agglutinin disease; PCH, paroxysmal cold hemoglobinuria. ^#^ Direct antiglobulin test; * Rarely due to IgM or IgA antibodies; ^+^ Agglutination titer can be high at colder temperatures (≤RT), may react up to ≤30 °C, usually anti-I/i specificity; ** Diagnosis made using Donath-Landsteiner (D-L) Test, usually anti-GLOB, fails to react with p RBCs; *** IgG can be found in the DAT using special tests but not with standard serological tests or in eluates using standard methods; ^ Can be positive with C3d; ^&^ Usually has specificity within ABO (anti-A or anti-B) or Rh (anti-D) blood group system.

**Table 3:** Summary of Pathophysiology and Treatments.

Category	Pathophysiology	Primary Target Antigen	DAT as Biomarker	First-Line Treatments ^&^
wAIHA	Extravascular hemolysis *	Band 3	IgG ± C3d	Glucocorticoids, steroid-sparing agents ^#^
CAS	Intravascular hemolysis **	I/i antigens	C3d only	Keep warm
CAD	Intravascular hemolysis **	I/i antigens	C3d only	Anti-C1q/-C5
Mixed AIHA	Intra- and extravascular hemolysis	Band3; I/i antigens	IgG + C3d	wAIHA, CAS, CAD options
PCH	Intravascular hemolysis	GLOB (formally P antigen)	C3d	Anti-C1q/-C5, Rituximab
DAT-neg AIHA	Extravascular hemolysis	Unk	negative	wAIHA options
DIAIHA	Intra-and extravascular hemolysis	Unk ^	IgG and/or C3d	Discontinue drug
PLS	Extravascular hemolysis	ABO/Rh	IgG	antigen-negative transfusion support; may adjust anti-rejection regimen

DAT, direct antiglobulin test; wAIHA, warm-antibody autoimmune hemolytic anemia; CAS, cold agglutinin syndrome; CAD, cold agglutinin disease; PCH, paroxysmal hemoglobinuria; DIAIHA, drug-induced autoimmune hemolytic anemia; PLS, passenger lymphocyte syndrome. ^&^ Transfusion if necessary; do not withhold transfusion if physiologically required; incompatible transfused blood (emulating the recipient antigen profile) is expected to have survival similar to autologous blood; caveats relate to underlying alloantibodies, which may be detected in adsorption studies or anticipated by recipient antigen profiling (genotyping, or where feasible, phenotyping). Depths of matching (mirroring what the patient is antigen-negative for, at a minimum in the Weiner-Kell system, and ideally beyond this in the Kidd, Duffy, and S systems) are key to avoiding missed antibodies, preventing the formation of new specificities, and helping to reduce the workload of adsorption studies assessing for residual/emerging alloantibodies permitted by historic match gaps. * Mediated by monocyte-macrophage phagocytosis of IgG and/or complement opsonized RBCs. Possibly intravascular, mediated by antibody-dependent cellular cytotoxicity (ADCC). ^#^ First choice treatment; other treatments are available such as rituximab, daratumumab and kinase inhibitors (see Text). ** Mediated by complement activation. ^ Target antigen requires the presence of the drug (in vivo and/orin vitro).
